# Pickering Emulsion-Based Microreactors for Size-Selective Interfacial Enzymatic Catalysis

**DOI:** 10.3389/fbioe.2020.00950

**Published:** 2020-08-21

**Authors:** Jieqing Lei, Liang Qi, Zhigang Luo

**Affiliations:** ^1^School of Food Science and Engineering, South China University of Technology, Guangzhou, China; ^2^South China Institute of Collaborative Innovation, Dongguan, China; ^3^Overseas Expertise Introduction Center for Discipline Innovation of Food Nutrition and Human Health (111 Center), Guangzhou, China

**Keywords:** ZIF-8, self-assembly, microcapsules, enzyme immobilization, biocatalysis, size selectivity

## Abstract

In this study, we have developed a mild and effective method to prepare a metal-organic framework (MOF)-based microcapsule by the self-assembly of pre-synthesized zeolite imidazolate framework-8 (ZIF-8) nanoparticles at the oil-water interface combined with deposition of a dense ZIF-8 coating outside the capsule. By introducing the enzyme *Candida antarctica* lipase B (CalB) directly into the stabilizer ZIF-8 or the water phase of Pickering emulsion during the preparation process, we achieved that the enzyme was immobilized within the shell (CalB@ZIF-8@cap) or in the cavity (ZIF-8@cap-CalB) of the microcapsules, respectively. The resulting CalB-loaded microcapsules were robust and had a core-shell structure proved by scanning electron microscopy. Meanwhile, Fourier transform infrared spectroscopy was conducted to confirm the encapsulation of enzymes in the microcapsules and their position in the microcapsules was confirmed by fluorescence microscopy. Furthermore, through the comparison of transesterification reactions between a pair of small substrates and a pair of larger ones, the two types of CalB-loaded microcapsules showed great catalytic activity, stability and size selectivity, and the catalytic activity of CalB@ZIF-8@cap was slightly higher than that of ZIF-8@cap-CalB. Importantly, due to the large size of the microcapsules, the catalyst could be separated from the reaction system by sedimentation, thereby reducing the energy consumption for separation. These kinds of multifunctional MOF-enzyme composites may open up new opportunities for the biocatalysis and microreactor.

## Introduction

Enzymatic catalysis is one of the important ways to achieve green and sustainable industrial chemical processes. However, free enzymes are usually subjected to some inherent shortcomings, such as fragile structures, low operational stability, and lack of reusability (Du et al., 2017). Therefore, it is of great importance to immobilize enzymes on solid carriers to improve their stabilities and achieve their facile recoveries during industrial applications (Duan et al., 2019). To date, significant efforts have been devoted to immobilize enzymes onto various carriers and composite materials, such as hydrogels (Jo et al., 2019), mesoporous silica (Popat et al., 2011; Li et al., 2019), magnetic nanoparticles (Desai and Pawar, 2020), polymers (Kuiper et al., 2008), and proteins (Kim et al., 2019).

In recent years, metal-organic frameworks (MOFs), a kind of porous crystalline organic–inorganic hybrid materials, composing of inorganic metal ions or metal clusters and organic ligands, have emerged as an excellent carrier for enzyme immobilization because of their fascinating physicochemical properties like thermal stability, high-surface area, pore size selectivity, and biocompatibility (Tan et al., 2019; Zhao et al., 2019; Zhou et al., 2019). Several approaches including physical adsorption (Nobakht et al., 2018; Jia et al., 2019), covalent bonding (Yue et al., 2016; Nowroozi-Nejad et al., 2019), and biomimetic mineralization (Liang et al., 2015; Jiang et al., 2017) have been employed to immobilize enzymes into MOFs. Among these methods, physical adsorption relies on numerous weak interactions (e.g., electrostatic interactions, hydrogen bonding, and van der Waals forces) between enzyme molecules and MOF crystals (Drout et al., 2019), thus adsorbed enzymes usually reveal poor stability in a harsh environment. Covalent binding is a great way of preventing enzymes from leaching, but it often induces the enzyme irreversibly deactivation during the preparation (Garcia-Galan et al., 2011).

Biomimetic mineralization allows MOFs to grow around enzymes in a mild reaction condition. The high chemical and structural stability of MOFs can not only protect the enzyme from various denaturation factors but regulate the access of substrates with different sizes (Wu et al., 2017). However, it has been found that the introduction of enzymes in MOFs has certain impacts on the structural characteristics of MOF inevitably. Qi et al. (2019) showed that the size of MOF nanoparticles loaded with lipase from *Candida rugosa* (CRL) was 2–3 times smaller than that of pure MOF nanoparticles. Therefore, recycling enzyme-MOF composites are hampered because the existing methods for separation, such as filtration and centrifugation, are obviously high time- and energy-consumption especially when the catalyst sizes are in the nanometer range. Meanwhile, enzymes exposed to the surface of MOF nanoparticles increased the hydrophilicity of enzyme-MOF composites. Thus enzyme-MOF composites are more likely to aggregate together in the organic reaction system, which is not conducive to fully exhibit the catalytic activity.

Increasing the size of enzyme-MOF composites is an effective way to improve its separation efficiency and distribution uniformity in solvents. So far, crosslinking is the most common method. The chemical modification of enzymes or MOFs are necessary steps for facile crosslinking, however it apparently has some negative effects on the efficiency of enzymatic reaction (Mehde, 2019; Oliveira et al., 2019). Moreover, because of the irregular congregation, the specific surface area of enzyme-MOF composites is significantly reduced after crosslinking.

Recently, a few efforts have been devoted to preparing microcapsules for efficient catalysis (Huo et al., 2015; Zhu et al., 2018). The pre-synthesized nanosized catalysts were assembled into microcapsules by self-assembly in Pickering emulsion. The resulting microcapsules with a core-shell structure were robust, high specific surface area and resizable by controlling the size of emulsion droplets. More importantly, the microcapsules have been demonstrated to be powerful and efficient bioreactors by encapsulation and attachment of enzymes (Wang et al., 2012; Liu et al., 2015). Therefore, constructing an enzyme-MOF composite based microcapsule is a promising way to increase the size of catalyst while maintaining the enzyme activity, size selectivity and specific surface area to the maximum extent.

Zeolitic imidazolate frameworks (ZIFs) are a subclass of MOFs with zeolite topologies (Fu et al., 2019). Among various ZIF materials, ZIF-8 is one of the most applied ZIF materials due to its high stability in water, and it also can be synthesized in water at room temperature, which is of great significance for protecting the activity of the enzyme during the enzyme immobilization (Horcajada et al., 2012).

In this work, we developed a facile strategy to construct a MOF based microcapsule by the self-assembly process of ZIF-8 nanoparticles in the oil-water surface of Pickering emulsion, followed by depositing a layer of small-sized ZIF-8 nanoparticles around emulsion droplets to enhance capsule stability. By introducing the model enzyme *Candida antarctica* lipase B (CalB) directly into the ZIF-8 or the water phase of ZIF-8 stabilized Pickering emulsion during the preparation process, we achieved that the enzyme was immobilized in the cavity or within the shell of the microcapsules, respectively. The biocatalytic reaction proved that the synthesized MOF-enzyme composites had excellent catalytic ability and size selectivity. Furthermore, due to the significantly increased size of the catalyst, the microcapsules could be quickly separated from the reaction system by natural sedimentation after the reaction.

## Materials and Methods

### Materials

Zinc acetate [Zn(Ac)_2_⋅2H_2_O, 99.0%], 2-Methylimidazole (2-Melm, 98%), 2,2′-Biquinoline-4,4-dicarboxylic acid disodium salt (BCA), vinyl acetate, 3-(4-hydroxyphenyl) propan-1-ol and Fluorescein isothiocyanate isomer I (FITC) were all purchased from Aladdin (Shanghai, China). Lipase B *Candida Antarctica* recombinant from *Aspergillus niger* (CalB) and agarose (Type IX-A, ultra-low gelling temperature) were purchased from Sigma-Aldrich (Shanghai, China). Other chemicals were of analytical grade and obtained from commercial sources. Pickering emulsions were formed using a shear force created by an IKA Ultra-Turrax T25 Homogenizer. Lyophilized samples were obtained by using a Scientz-10N freeze-dryer.

### Characterization

Optical microscopy (OPM) measurements were carried out on a MB11 polarizing microscope (Shanghai optical instrument factory, China) equipped with a video camera.

UV-vis adsorption was acquired on an ultraviolet-visible spectrophotometer (Hitachi U-3010, Japan).

Powder X-ray diffraction (XRD) patterns were collected using a D/max-IIIA fully automatic XRD instrument (Rigaku, Japan) with a Cu Kα anode (λ = 1.5405 Å) at 40 kV and 30 mA. The powdered samples were scanned from 5 to 40° (2θ) at a scanning rate of 4°/min.

^1^H-NMR spectra were obtained using an AVANCE digital 400 (Bruker, Germany) operating at 400 MHz.

Scanning electron microscopy (SEM) measurements were made on a Merlin scanning electron microscope (Zeiss Co., Germany). Samples for SEM measurements were firstly dispersed in absolute ethanol (0.1 wt%) and sonicated for 10 min. A drop of suspension was carefully placed on an aluminum foil attached to a substrate with conductive tape and dried at room temperature, and then sputter-coated with a thin layer of conductive gold to improve conductivity.

Fourier transform infrared (FTIR) spectroscopy was carried out on a Vector 33-MIR FTIR spectrophotometer (Brukev Optik, Germany) using the KBr method.

The presence and spatial location of the fluorophore-tagged enzymes in the microcapsules was determined with an IX73 inverted fluorescence microscope (OLYMPUS, Japan).

### Synthetic Details

#### Preparation of CalB@ZIF-8 and ZIF-8

160 μL of CalB were added into a solution of 2-Melm (1.25 M, 40 mL) in deionized water. A separate solution of Zn(Ac)_2_⋅2H_2_O (0.31 M, 4 mL) was also prepared. These two solutions were mixed and then stirred at 500 rpm for 8 h at room temperature. Then the products (named as CalB@ZIF-8) were collected by centrifugation at 10,000 rpm for 10 min and washed with deionized water for three times to remove the absorbed proteins. Finally, the centrifuged samples were collected after lyophilization.

The synthesis of pure ZIF-8 nanoparticles followed the same procedure as the preparation of CalB@ZIF-8 but in the absence of CalB.

#### Preparation of Pickering Emulsion

CalB was located within the shell of the microcapsule (CalB@ZIF-8@cap): 30 mg of CalB@ZIF-8 nanoparticles were added to 3 mL of medium chain triglycerides (MCT) and homogenized for 1 min to evenly disperse. Then 1.0 wt% agarose solution was prepared by dissolving 0.010 g of agarose in 1 mL of PBS buffer (pH = 7.4) at 80°C as stock solution. After cooling to 25°C, 200 μL of agarose solution was added to the MCT dispersion of CalB@ZIF-8 and homogenized under shear stirring at 16,000 rpm for 2 min. The resulting water-in-oil Pickering emulsion was stored at 4°C overnight to ensure that the nanoparticle-stabilized droplets were fully gelled.

CalB was located in the cavity of the microcapsule (ZIF-8@cap-CalB): the preparation method of water-in-oil Pickering emulsion was the same as above except that the pure ZIF-8 nanoparticles were applied to stabilize Pickering emulsion and CalB was added to the agarose solution. Notably, the enzyme was added after the agarose solution was cooled to 30°C to prevent the high temperature from affecting the enzyme activity.

#### Preparation of Microcapsules

First of all, 2 × 100 mL stock solutions of Zn(NO_3_)_2_ (25 mM) were prepared in isopropanol and 2-butanol, and 2 × 100 mL stock solutions of 2-Melm in isopropanol (100 mM) and 2-butanol (75 mM).

Then 2.5 mL of Zn(NO_3_)_2_ and 2-MeIm stock solution in isopropanol were stepwise added into 1 mL of agarose solution droplets and left at −20°C for 2 h before washing with fresh isopropanol. After repeating the above procedure twice, the obtained microcapsules were immersed in a fresh mixture of 2.5 mL Zn(NO_3_)_2_ stock solution and 2.5 mL 2-MeIm stock solution in 2-butanol. The mixture was left for 1.5 h at −20°C before washing with fresh 2-butanol. This step was repeated seven times to ensure a dense shell of small-sized ZIF-8 nanoparticles at the capsule exterior.

#### Calculation of Enzyme Loading

Enzyme loading capacity of CalB@ZIF-8 nanoparticles and CalB-loaded microcapsules were calculated by the BCA method. Specifically, 10 mg of sample was ground with a mortar and then destroyed by adding 6 mL of 0.2 M HCl aqueous solution and 40 μL of HF (48% water solution), followed by centrifugation at 10,000 rpm for 10 min. After filtering the supernatant through a 0.22 μm filter, a 0.2 mL of sample was taken and mixed with 2 mL of BCA reagent, which was incubated at 37°C for 30 min prior to UV measurement. To minimize potential errors caused by the low pH, degraded pure ZIF-8 nanoparticles were applied for standardization. The difference of intensity at 562 nm of the sample and the standard was used to calculate the concentration of CalB within the CalB@ZIF-8 nanoparticles and microcapsules.

The calculation of enzyme loading is based on Eq. (1)

(1) $$\mathrm{Enzyme}\,\mathrm{loading}\,\left(\text{mg}\cdot {\text{g}}^{-1}\right)=\frac{C\,V}{m}$$

where m (g) is the total amount of CalB@ZIF-8 nanoparticles or CalB-loaded microcapsules; C (mg⋅mL^–1^) and V (mL) are the CalB concentration and the volume of the supernatant, respectively.

#### Biocatalysis

The activity of CalB was determined by the transesterification between two groups of substrates in n-heptane: small substrate, 1-butanol and vinyl acetate; large substrates, 3-(4-hydroxyphenyl)propan-1-ol and vinyl laurate. Specifically, as for small substrates, two types of CalB-loaded microcapsules and CalB@ZIF-8 nanoparticles with the same enzyme loading were added into a substrate solution (3 mL) containing 1-butanol (150 mmol/L) and vinyl acetate (100 mmol/L) in n-heptane. Then the reactions were carried out on a shaker at 35°C for a total of 48 h. After a defined interval, a 60 μL sample of the solution was removed and 1H-NMR was conducted to analyze the concentration of the product. One unit of CalB activity (U) is defined as the enzyme amount needed for producing 1 μmol product per minute. The activity of all samples was tested under the same conditions and all reactions were repeated at least three times. The reaction of large substrates followed the same procedure.

#### Catalytic Recyclability

The recoverability of the catalyst was evaluated by measuring the conversion of each cycle. After each batch, the microcapsules were collected through centrifugation (800 rpm) and then washed with n-heptane, and utilized for the next cycle. The maximum conversion rate was defined as 100%, and the relative conversion (%) represented the ratio of residual to maximum conversion rate of each sample.

## Results and Discussion

**SCHEME 1:**
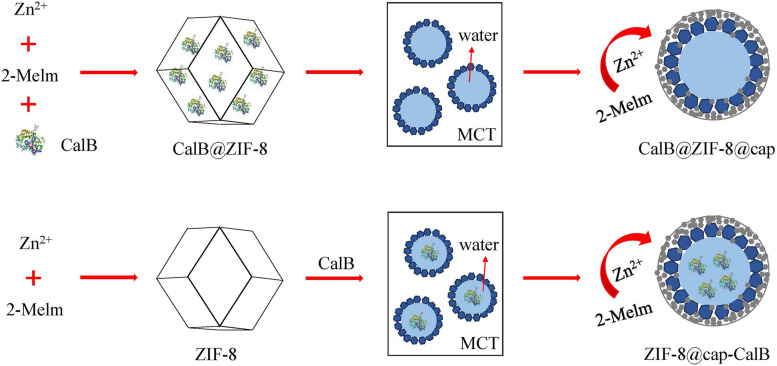
Preparation of two types of CalB-loaded microcapsules.

In this study, two different methods were used to introduce CalB into the ZIF-8 based microcapsules. The first strategy was to encapsulate the enzyme in the cavity of ZIF-8 nanoparticles and followed by formation of the Pickering emulsion based microcapsules (CalB@ZIF-8@cap). The second strategy was to directly dissolve the enzyme in water phase and then formed Pickering emulsion based microcapsules (ZIF-8@cap-CalB). In particular, to enhance the stability of both microcapsules, the deposition of a layer of small-sized ZIF-8 nanoparticles was necessary outside each microcapsule (Scheme 1).

### ZIF-8 and CalB@ZIF-8

**FIGURE 1:**
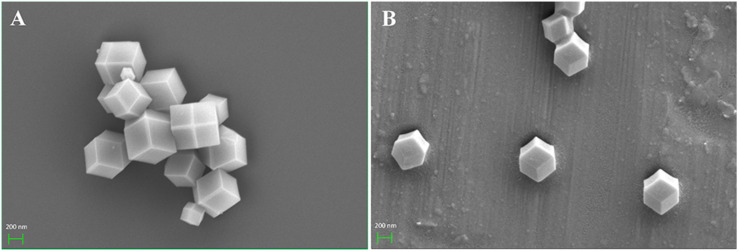
SEM images of **(A)** ZIF-8 and **(B)** CalB@ZIF-8 nanoparticles.

In this study, ZIF-8 and CalB@ZIF-8 nanoparticles were prepared via self-assembly and biomimetic mineralization methods as reported by us (Scheme 1; Qi et al., 2019). The SEM images showed that both ZIF-8 and CalB@ZIF-8 nanoparticles presented rhombic dodecahedron, indicating that the encapsulation of the enzyme did not significantly affect the morphology of ZIF-8 nanoparticles. However, the particle size of CalB@ZIF-8 nanoparticles (∼500 nm) was smaller than that of ZIF-8 (∼700 nm), which proved that the introduction of enzymes would reduce the size of ZIF-8 nanoparticles (Figure 1).

### CalB-Loaded Microcapsules

**FIGURE 2:**
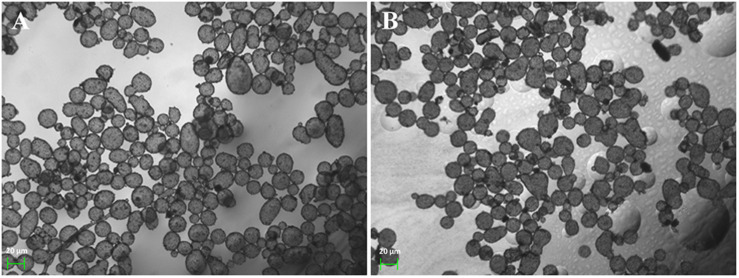
Optical microscopy images of **(A)** CalB@ZIF-8@cap and **(B)** ZIF-8@cap-CalB.

**FIGURE 3:**
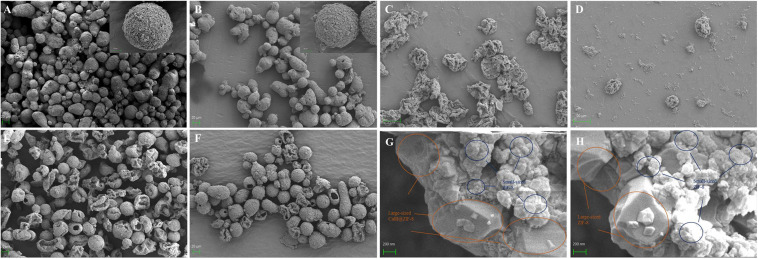
Characterization of MOF-based microcapsules. The SEM images of the synthesized intact **(A)** CalB@ZIF-8@cap and **(B)** ZIF-8@cap-CalB. **(C)** CalB@ZIF-8@cap and **(D)** ZIF-8@cap-CalB without dense layers outside the shell. **(E,G)** The crushed sample of CalB@ZIF-8@cap, showing their hollow core-shell structure and hierarchically structured shell. **(F,H)** The crushed sample of ZIF-8@cap-CalB, showing their hollow core-shell structure and hierarchically structured shell.

Since both microcapsules were acquired through the self-assembly of pre-synthesized nanoparticles in the oil–water interface and followed by deposition of a layer of small-sized ZIF-8 nanoparticles outside capsules, CalB@ZIF-8@cap and ZIF-8@cap-CalB had the similar morphology. Under the optical microscope, the both microcapsules were observed to be round or oval, with a uniform size ranging from 20 to 40 μm (Figure 2). The SEM pictures clearly showed that both two types of microcapsules were intact sphere, whose surface was covered by a layer of dense small-sized ZIF-8 nanoparticle, and remained unbroken even after the drying process (Figures 3A,B). In contrast, the microcapsules without a layer of small-sized ZIF-8 nanoparticles exhibited the vulnerable structure and easily collapsed after drying (Figures 3C,D). It strongly demonstrated that small-sized ZIF-8 nanoparticles which grew on the surface of the microcapsules during the deposition process brought additional structural stability to the microcapsules.

After the formed spheres were ground well with a mortar, the broken spheres of both types of microcapsules revealed the hollow interior, confirming the microcapsules structures (Figures 3E,F). The cross section of capsules shell had a clear hierarchical structure and was divided into two layers. The interior of the shell was composed of a continuous network of aggregated large-sized ZIF-8 or CalB@ZIF-8 nanoparticles which were used to stabilize Pickering emulsion. The outside of the shell was a dense coating deposited by small-sized ZIF-8 nanoparticles (Figures 3G,H).

**FIGURE 4:**
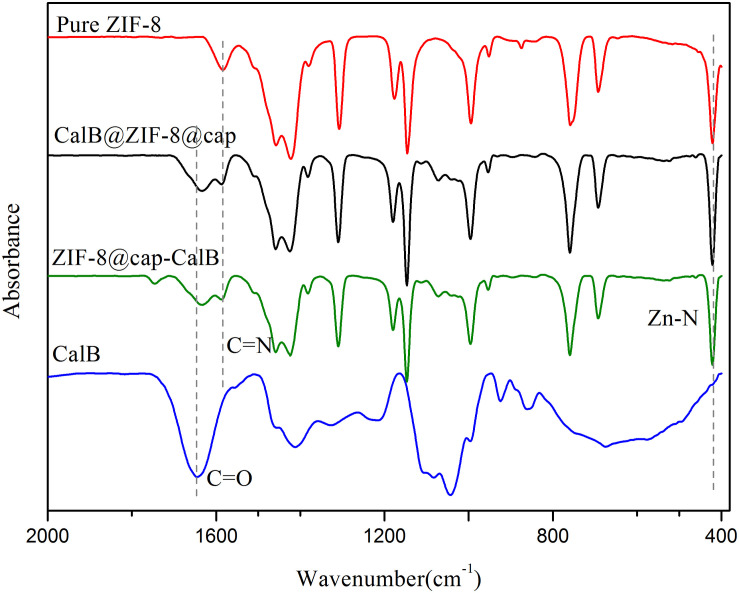
FTIR spectra of pure ZIF-8, CalB, ZIF-8@cap-CalB, and CalB@ZIF-8@cap.

**FIGURE 5:**
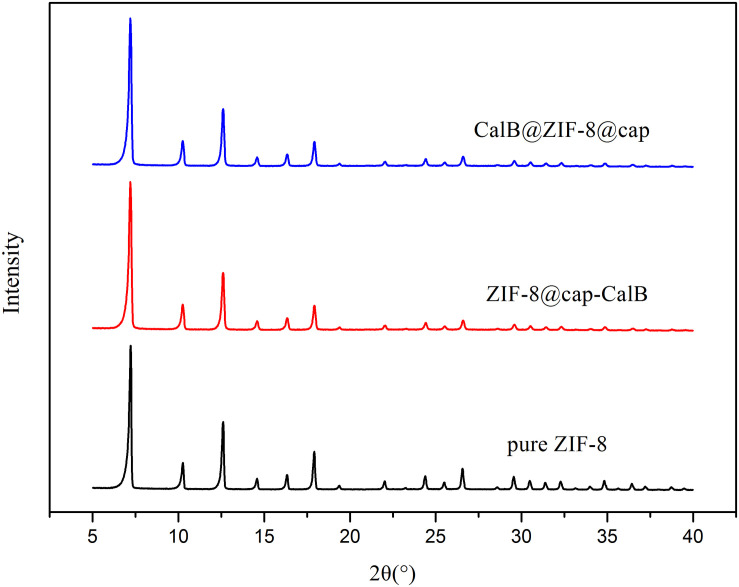
XRD patterns of pure ZIF-8, ZIF-8@cap-CalB, and CalB@ZIF-8@cap.

Through these two methods, the enzyme could be immobilized in the cavity or within the shell of microcapsules. To further ascertain this, FTIR measurements of pure ZIF-8, CalB, CalB@ZIF-8@cap, and ZIF-8@cap-CalB were conducted. As shown in Figure 4, the characteristic adsorption bands of ZIF-8 were observed at 1581 and 421 cm^–1^, ascribed to C = N stretching vibration on the imidazole ring and Zn-N stretching vibration. The characteristic absorption peak for CalB at 1640–1660 cm^–1^, which corresponded to amide I mainly from C = O stretching mode, was also found in spectra of CalB@ZIF-8@cap and ZIF-8@cap-CalB. In this study, it could be observed that there was a slight shift of the amide I peak in the CalB-loaded microcapsules, which could attribute to the direct protein-MOF interaction caused by the coordination between Zn^2+^ and the carbonyl groups on the proteins. Besides, the decrease in peak intensity may be related to the amount of CalB (Qi et al., 2019). These data clearly confirmed the existence of CalB whether it located within the shell or in the cavity of the microcapsule. In addition, XRD patterns were also conducted (Figure 5). The sharpness and high intensity of the diffraction peaks indicated the high crystallinity of the nanoparticles. Meanwhile, CalB@ZIF-8@cap and ZIF-8@cap-CalB presented similar X-ray diffraction patterns with the pure ZIF-8 nanoparticles, suggesting encapsulation of CalB hardly affected the crystal structure of ZIF-8.

**FIGURE 6:**
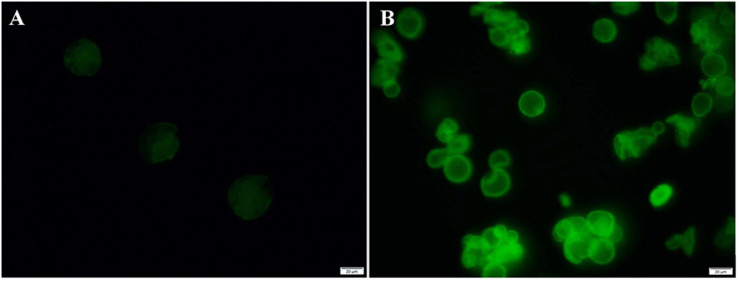
Fluorescence analysis of FITC labeled microcapsules. The fluorescence microscopy images of **(A)** FITC labeled ZIF-8@cap-CalB and **(B)** FITC labeled CalB@ZIF-8@cap.

Before performing the catalytic experiments, the fluorescence microscopy images were taken with FITC-labeled CalB to further determine the distribution of enzymes in the microcapsules. In Figure 6, the fluorescence microscopy images of FITC-labeled microcapsules showed strong fluorescence intensity (λ_ex_ = 488 nm). The reflection image was attributed to the reflected light of fluorescent molecules at the spherical surface (Xu et al., 2018). For FITC-labeled ZIF-8@cap-CalB, the fluorescence image of FITC-labeled microcapsules was negligible, which indicated that CalB was not on the surface of microcapsules and thus they were retained within microcapsules (Figure 6A). In contrast, since the enzyme was located within the shell of the CalB@ZIF-8@cap, the FITC reflection was obvious (Figure 6B).

### Biocatalysis

CalB is a highly versatile lipase due to its broad substrate selectivity and catalytic functionality. It can be used to catalyze a variety of reactions including aminolysis, esterification, transesterification and hydrolysis, and is stable over a wide range of biophysical conditions including pH, temperature and solvent conditions, making it an excellent model enzyme for encapsulation and biocatalytic studies (Monteiro et al., 2019; Van Tassel et al., 2020; Zhang et al., 2020). Two kind of CalB-loaded microcapsules were obtained as described previously. After degrading the microcapsules by acid, the CalB loading was calculated by Bradford assay. The results showed that the enzyme loading of the first method (CalB@ZIF-8@cap) was 3.96 mg⋅g^–1^, and that of the second method (ZIF-8@cap-CalB) was 3.52 mg⋅g^–1^.

**FIGURE 7:**
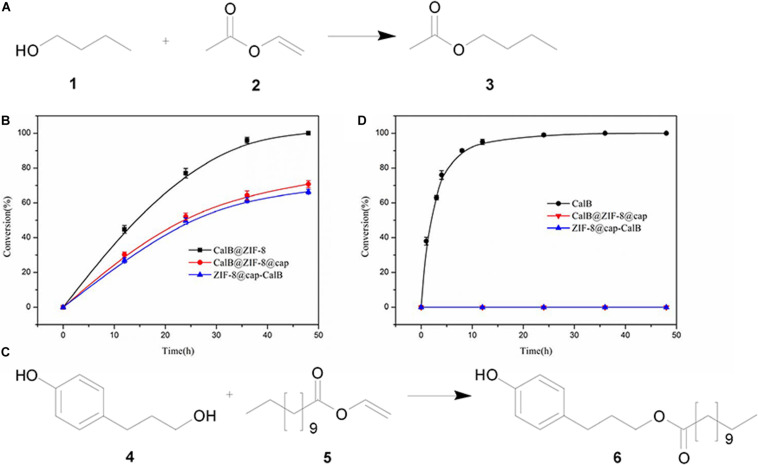
Biocatalysis using CalB loaded microcapsules. **(A)** Reaction scheme for the transesterification between 1-butanol (1) and vinyl acetate (2) to yield butyl acetate (3). **(B)** Time-dependent conversion of 1 and 2 catalyzed by CalB under different conditions. **(C)** Reaction scheme of transesterification between 3-(4-hydroxyphenyl) propan-1-ol (4) and vinyl laurate (5) to yield 3-(4-hydroxyphenyl)propyl dodecanoate (6). **(D)** Time-dependent conversion of 3 and 4 catalyzed by CalB under different conditions.

**FIGURE 8:**
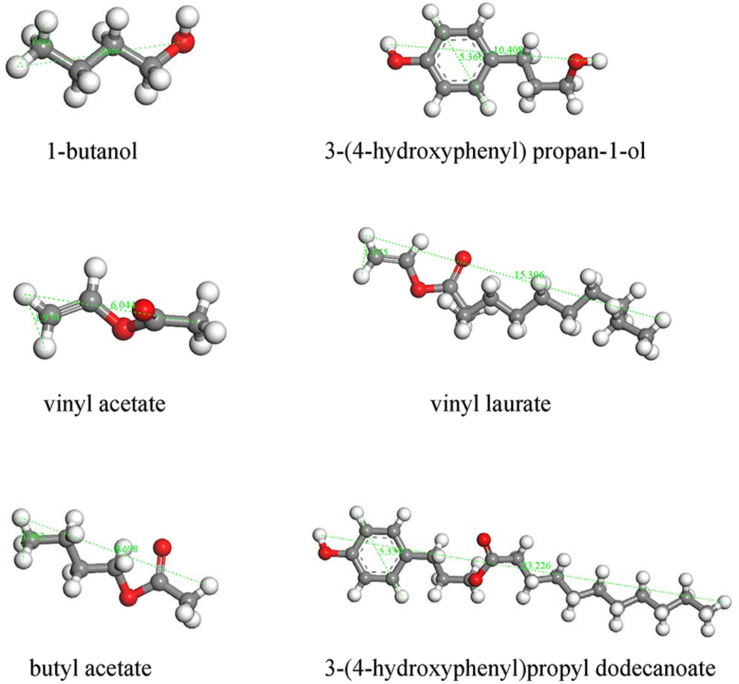
Molecular model of the substrates used and products formed following transesterification with CalB.

The catalytic performances of the two types of CalB-loaded microcapsules were investigated by carrying out transesterification reactions between a pair of small substrates as shown in Figure 7A. In this reaction, 1-butanol and vinyl acetate were catalyzed by lipase to produce butyl acetate. Both the substrates and the products of this reaction had a small size, and theoretically could be diffused through the micropores of ZIF-8 nanoparticles (Figure 8). CalB@ZIF-8 nanoparticles were also carried out as positive control. Catalysis reactions were carried out at 35°C in n-heptane, a shaker was implied to ensure adequate contact between the substrates and the catalyst. The catalytic experiments were performed for a total of 48 h, the samples were taken at regular intervals to calculate the substrates conversion rate by ^1^H-NMR to evaluate the enzyme activity.

The results showed that the positive control took about 48 h to reach 100% conversion, while both of CalB-loaded microcapsules reached a conversion rate of about 70% at the same time (Figure 7B). These data indicated that the enzyme activity was maintained well during the formation of the microcapsules and the substrates and products could diffused through the framework micropores of the intact shell. However, the catalytic efficiency of the two kinds of CalB-loaded microcapsules were lower than the free CalB@ZIF-8 nanoparticles, which may be due to the time required for the substrates and products to pass through the dense shell of the microcapsules.

**FIGURE 9:**
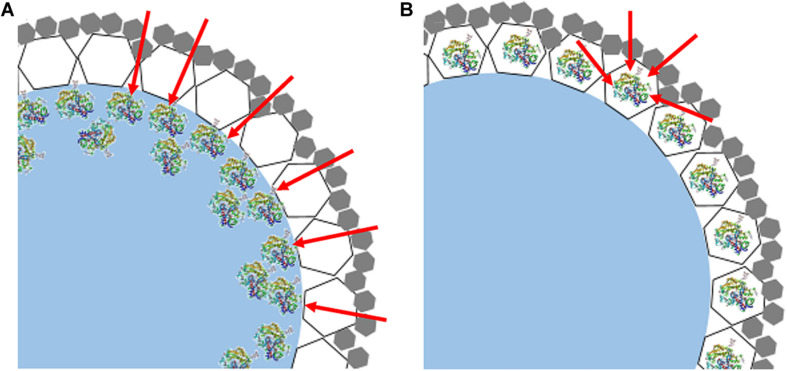
Model diagrams of the cross section of the shell of **(A)** ZIF-8@cap-CalB and **(B)** CalB@ZIF-8@cap. The white hexagons represent the large-sized ZIF-8 or CalB@ZIF-8 used to stabilize the Pickering emulsion. The gray hexagons represent the deposited small-sized ZIF-8 coating.

Meanwhile, it was noticed that ZIF-8@cap-CalB has slightly lower catalytic efficiency than CalB@ZIF-8@cap. It was because in the first case, most of the enzyme molecules located in the cavity of the microcapsules were adsorbed and accumulated on the inner wall of the microcapsules, which was most likely to be surface adsorption caused by interactions between the abundant organic functional groups of the MOF particles and the enzyme (Huo et al., 2015). Therefore, the aggregation of the enzyme allowed the substrates to contact the enzyme only at the interface, thereby greatly reducing the effective contact area of the catalytic reaction (Figure 9A). However, in the second case, most of the enzyme molecules located within the microcapsule shell were evenly distributed in the ZIF-8 nanoparticles, so the substrates could contact the enzyme from all directions of the ZIF-8 nanoparticles (Figure 9B). Therefore, the effective contact areas between the enzyme and the substrates in the second case were significantly higher than that in the first case. Meanwhile, the hierarchical structure of the microcapsule shell significantly increased the time for substrates to pass through the shell, thereby inducing more substrates participating in the reaction. These might be the reason why the catalytic efficiency of CalB@ZIF-8@cap was higher than ZIF-8@cap-CalB.

**FIGURE 10:**
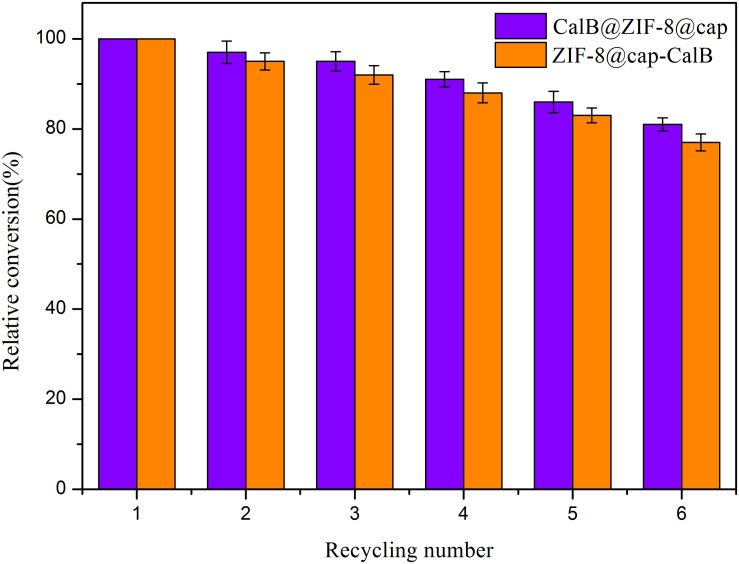
Recycling tests of CalB@ZIF-8@cap and ZIF-8@cap-CalB for the transesterification between a pair of small substrates.

When finished, due to the large size of the microcapsules, the catalysts could be easily separated from the reaction system by natural sedimentation. Compared with traditional catalysts separated by high-speed centrifugation, a large amount of energy input was saved, which was more in line with the requirements of green and sustainable industrial development. The easy of recovery prompted us to investigate the catalytic recyclability of these two types of microcapsules. Following transesterification reaction between a pair of small substrates for 8 h, CalB@ZIF-8@cap and ZIF-8@cap-CalB were separated, washed and used again for further reaction. Conversion of butyl acetate revealed very good overall recyclability for both microcapsules with ∼80% of activity maintained after six catalytic cycles (Figure 10). This showed that the catalyst was not only easy to separate from the reaction system, but also had good catalytic recyclability.

The shells of both CalB-loaded microcapsules were deposited with a dense ZIF-8 coating. Due to the limitation of the pore size of ZIF-8 nanoparticles (3.4 Å) (Schejn et al., 2014), there would be a size selection effect on the substrates catalyzed by the enzymes in both CalB-loaded microcapsules. To verity the size selectivity of the two types of CalB-loaded microcapsules, a transesterification reaction between a pair of large substrates, 3-(4-hydroxyphenyl) propan-1-ol and vinyl laurate, was also performed (Figure 7C). The results showed that when the reaction was catalyzed by free CalB, the conversion reached 100% after 24 h, while when catalyzed by the two types of CalB-loaded microcapsules, almost no product was formed after 48 h (Figure 7D). This indicated that almost no substrates passed through the micropores to react with the enzyme, and the synthesized microcapsules could regulate the access of substrates with different sizes.

## Conclusion

In summary, we have developed a simple and effective method to prepare large-sized and easily separable catalysts, which were made by the self-assembly of pre-synthesized ZIF-8 nanoparticles at the oil-water interface. At the same time, we have achieved the immobilization of enzymes at different positions in the microcapsules (in the cavity of the microcapsule or within the shell of the microcapsule) and demonstrated their practical value in terms of biocatalysis and size selectivity. Compared with traditional enzyme@MOF catalyst, the synthesized enzyme-loaded microcapsules with a hierarchical structure could increase the size of the catalyst from nanoscale to the micrometer scale and still retain a high catalytic activity, so that it could be separated from the reaction system by sedimentation. This is a very novel strategy for preparing large-size and multifunctional MOF-biomolecule composites, which has great potential in transportation, microreactor and biotechnology.

## Data Availability Statement

All datasets generated for this study are included in the article/supplementary material.

## Author Contributions

JL performed the experiments and wrote the manuscript. JL and LQ designed the experiments and interpreted the experimental data. ZL conceived the idea and supervised the whole research. All authors contributed to the article and approved the submitted version.

## Conflict of Interest

The authors declare that the research was conducted in the absence of any commercial or financial relationships that could be construed as a potential conflict of interest.
